# Development and characterization of a new cell line derived from European eel *Anguilla anguilla* kidney

**DOI:** 10.1242/bio.037507

**Published:** 2018-11-14

**Authors:** Bin Chen, Zaiyu Zheng, Jinxian Yang, Hongshu Chi, He Huang, Hui Gong

**Affiliations:** Biotechnology Institute, Fujian Academy of Agricultural Sciences, High-tech building 1506, Wusi Road 247, Fuzhou 350003, Fujian, China

**Keywords:** *Anguilla anguilla*, Kidney cell line, *Rana grylio* virus, Immune-related gene

## Abstract

A new cell line derived from the kidney of European eel, *Anguilla anguilla*, has been established and characterized. This cell line, designated as EK (eel kidney), has been maintained in Leibovitz's L-15 supplemented with 10% fetal bovine serum for over 24 months, and subcultured more than 60 times. This cell line consists predominantly of fibroblast-like cells, and can grow at 15–37°C under an optimum temperature of 26°C. The origin of this cell line was confirmed by polymerase chain reaction (PCR) amplification and *18s* recombinant (r)RNA sequencing. The chromosome analysis of EK cells at passage 58 revealed an ananeuploid karyotype. The EK cells were successfully transfected with the Pegfp-N1 plasmid, suggesting its potential in genetic studies. The susceptibility test showed a significant cytopathic effect (CPE) in EK cells for *Rana grylio* virus, and the viral replication was evidenced with quantitative real-time PCR (qRT-PCR) assay. After poly (I:C) stimulation, the expression of the immune-related molecules including interferon regulatory factor-3 (*irf3*), interferon regulatory factor-7 (*irf7*) and cytochrome P450 (*CYP*450) were significantly upregulated in EK cells, while the expression of transforming growth factor (TGF-*β*) was downregulated. These results suggested the potential of EK cell line as a model in gene engineering, virus identification and environmental toxicology.

## Abstract (precis)

**Summary:** The first visceral cell line of an endangered species, *Anguilla anguilla* has been established; this aneuploid cell line is fibroblast-like, and suitable for gene expression and virus isolation.

## INTRODUCTION

European eel *Anguilla anguilla* (L. 1758) was previously considered one of the most important aquaculture species in both Europe and China, but is now listed as endangered due to the threats of overfishing, diseases, obstacles, ocean current changes, polychlorinated biphenyl (PCB) pollution, etc. (de Boer et al., 1994; Dekker, 2004; van Ginneken, 2006; Hendriks et al., 2010; Knights, 2003). Both wild and farmed eels have suffered the attack of various viruses for over years, including herpesvirus, picornavirus and coronavirus (Fichtner et al., 2013; Ge et al., 2012, 2014; Jakob et al., 2009; van Beurden et al., 2012; Yue et al., 1998; Zhang and Gui, 2008).

Fish cell lines play an important role in the studies of aquatic virology, developmental biology, genetics, immunology, physiology, toxicology and pharmacology (Baksi and Frazier, 1990; Bols, 1991; Kohlpoth et al., 1999; Ni Shuilleabhaina et al., 2006). Since the setup of the first teleost cell line RTG-2 (Wolf and Quimby, 1962), over 300 fish cell lines have been established (Fryer and Lannan, 1994; Lakra et al., 2011). Dozens of viruses have been isolated using fish cell lines, and explorations in emerging fields such as immunological signaling, aquatic oligodynamics, genetic engineering and environmental monitoring have shown enormous prospects (Béjar et al., 2002; Bryson et al., 2006; Chen et al., 2005; Dong et al., 2008; Sahul-Hameed et al., 2006; Zhang et al., 2003).

To date, cell lines have been developed from only a few species of *Anguillidae* fishes, or in other words, the *Anguillidae* invitrome is small (Bols et al., 2017). The first cell lines were developed from the Japanese eel, *Anguilla japonica* (Temminck and Schlegel 1846) (Chen and Kou, 1988; Kou et al., 1995). More recently two cell lines, PBLE and eelB, have been described from the American eel, *A. rostrata* (Lesueur 1817) (Bloch et al., 2016; Dewitte-Orr et al., 2006). However, few cell lines have been reported from the European eel *A. anguilla* (Linnaeus 1758). In 2007, we initially tried *in vitro* tissue and cell culture of multiple European eel organs (Zheng, 2008). In this study, we have developed and characterized a cell line derived from *A. anguilla* kidney, which proved to be susceptible to *Rana grylio* virus (RGV). The responses of this cell line to regular immune stimulations were also investigated.

## RESULTS

### Primary cell culture and subculture

**Fig. 1.**
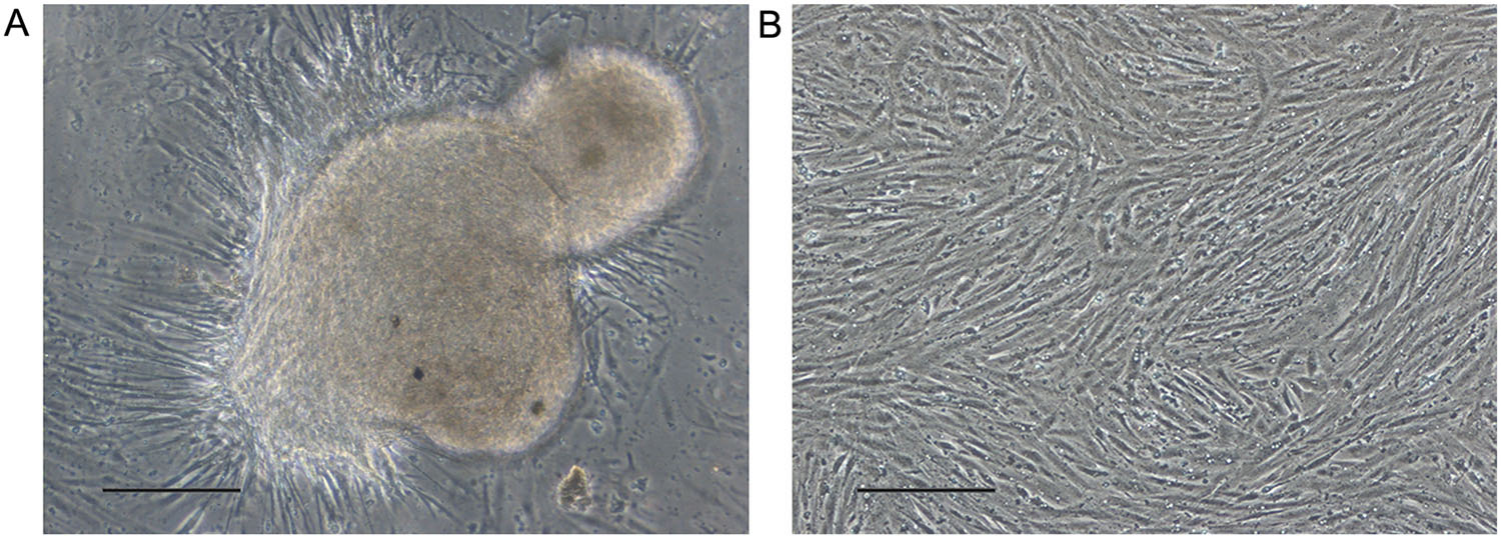
**Development of the *Anguilla anguilla* eel kidney cell line.** (A) The primary explant culture and cell migration. (B) The confluent culture of EK cells at passage 65, 36 h after inoculation. Scale bars: 50 μm.

After 24 h of inoculation, cells were migrated outwards from the tissue explants (Fig. 1A) and the first subculture was conducted on day 7. The subculture was performed at a split ratio of 1:2 every 36 h, and these cells were subcultured over 70 times to date. The eel kidney (EK) cell line was anchorage-dependent, predominantly made up of fibroblast-like cells (Fig. 1B) and was maintained in L-15 containing 10% fetal bovine serum (FBS) at 26°C. The EK cells recovered from liquid nitrogen storage at the 60th subculture – whose average viability was estimated to be 75%–85% – could reach confluency within 2 days.

### The growth studies

**Fig. 2.**
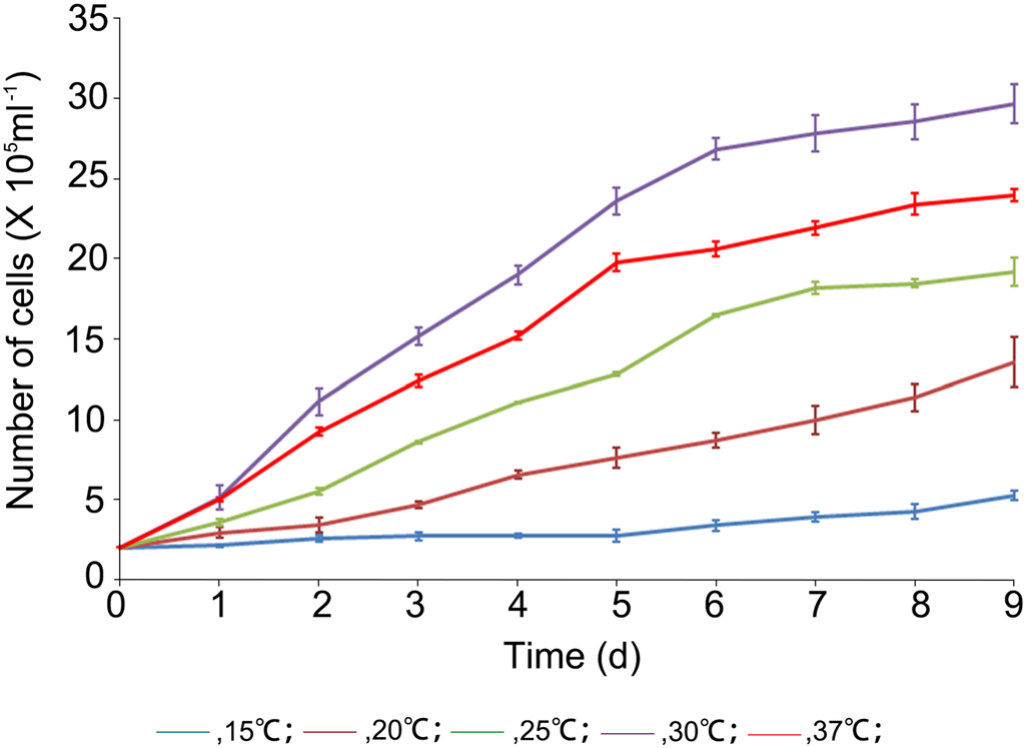
**The growth of EK cell line at different passages and temperatures.** Growth curves of EK cells at passage 63 (blue, 15°C; brown, 20°C; green, 25°C; purple, 30°C; red, 37°C). The maximum growth rate was obtained at 30°C. The values are displayed as mean±s.d. (*n*=3).

The EK cells grew into a confluent monolayer at a temperature range between 15°C and 37°C, and at 10°C or 40°C several small colonies were formed. The maximum growth rate was observed at 30°C (Fig. 2) 2–6 days after inoculation, and the passage 63 EK cells presented the logarithmic phase with a population doubling time (PDT) of 50.27 h.

### Chromosome analysis

**Fig. 3.**
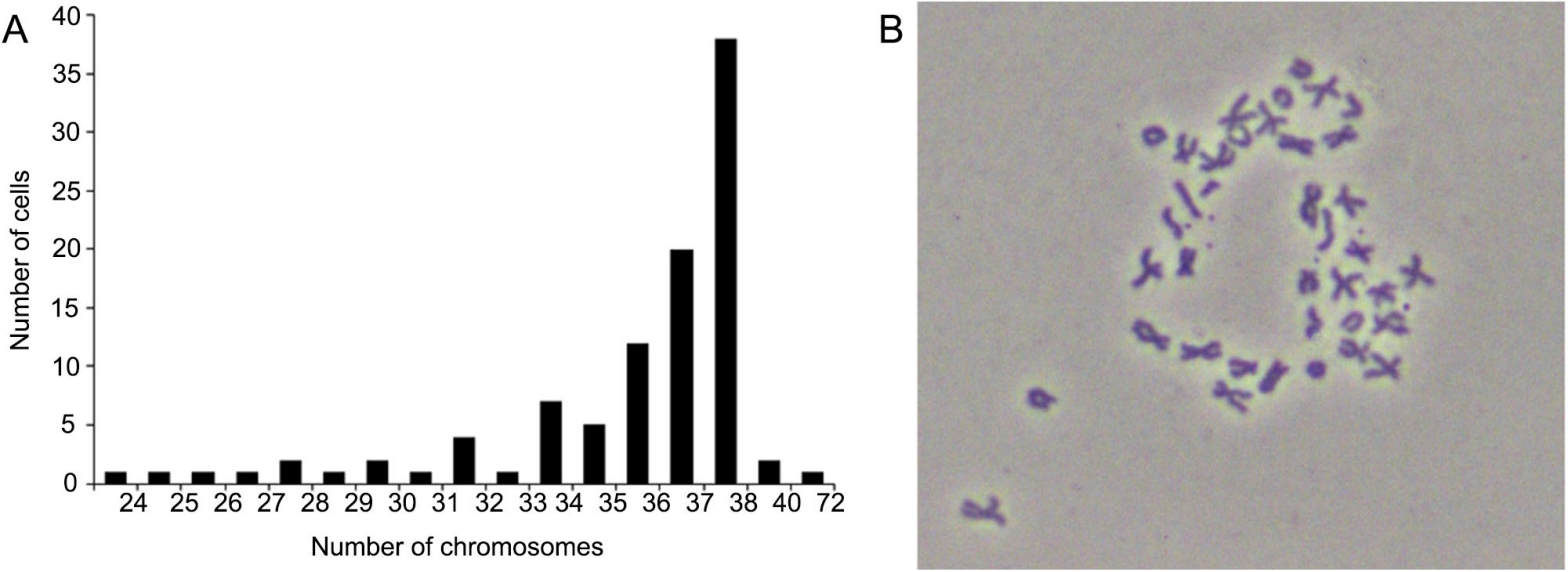
**Chromosome analysis of the EK cells at passage 58.** Chromosome number distribution (A) and metaphase (B) of the EK cells at passage 58. Thirty-eight percent of 100 samples presented chromosome number 38, which consisted of six pairs of mediocentrics, three pairs of subtelocentrics and 10 pairs of telocentrics (2n=6m+3st+10t), while the other 32% samples contained 36 or 37 chromosomes.

Among the 100 metaphase EK cells counted at passage 58, the chromosome numbers ranged from 24 to 72 (Fig. 3A). Thirty-eight percent of samples presented normal diploid karyotype of 2n=38; of these, the metacentric, submetacentric and telocentric chromosomes were 6, 3 and 10 pairs, respectively (2n=6m+3sm+10t) (Fig. 3B); while the remaining 32% samples contained of 36 or 37 chromosomes. These results suggested that EK may be an aneuploid cell line.

### Pegfp-N1 transfection

**Fig. 4.**
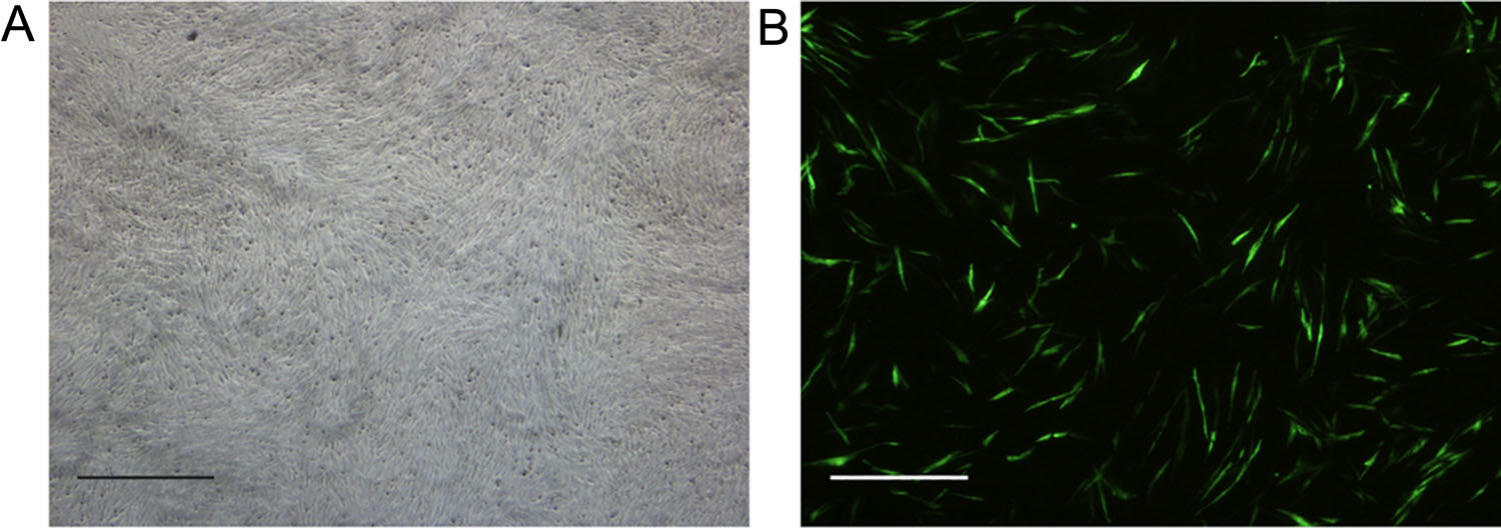
**EK cells transfected with pEGFP-N1 at passage 60.** (A) Optical microscope photograph and (B) the green fluorescence photograph of the EK cells transfected. The expression of GFP gene could be observed 24h after transfection. Scale bars: 50 μm.

Green fluorescence signals could be observed in the EK cells at 24 h after transfection, and lasted for 4 days or longer (Fig. 4A,B). The transfection efficiency was estimated to be about 10%.

### *18s* rRNA sequence analysis

The species of the EK cell line was confirmed by *18s* rRNA gene analysis. An expected, PCR product of 1702 bp was obtained using specific amplification of *18s* rRNA from the extracted total genomic DNA (Fig. S1), which was proved to be 100% identical to the published *A. anguilla 18s* rRNA sequence (GenBank: FM946070.1).

### Susceptibility test

**Fig. 5.**
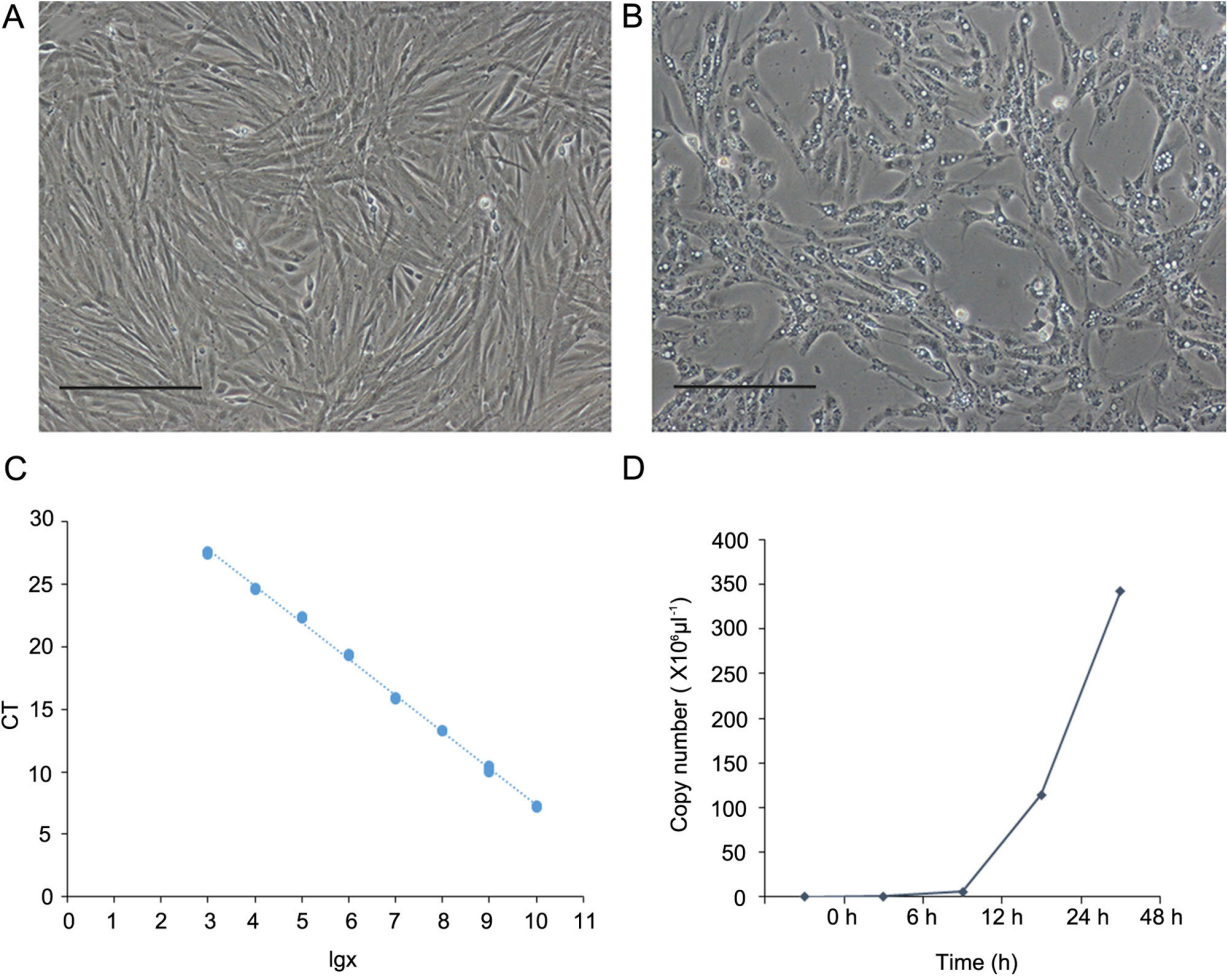
**RGV infection on EK cells at passage 45.** (A) Control cells at 24 h. (B) EK cells incubated at 26°C, 24 h after RGV inoculation, multiplicity of infection (MOI)=4.0. (C) Standard curve, y=−2.9141x+36.505, R^2^=0.9985, 3≤x≤10. (D) Number of RGV copies expressed in the EK cells. Scale bars: 50 μm.

Cytopathic effect was first observed at 24 h after infection, and was covered in over 75% of the monolayer at 48 h (Fig. 5B), while the monolayer in the controls stayed healthy (Fig. 5A). The qRT-PCR standard curve was plotted using linear-regression analysis according to the sequencing report of the pMD-19T-MCP vector: y=_−_2.914 x+36.505, R^2^=0.9985, 3≤x≤10 (Fig. 5C), transcripts of MCP were increased significantly in the EK cells from 6 to 48 h after infection with RGV (Fig. 5D).

### Immune-related gene expression after poly (I: C) exposure

**Fig. 6.**
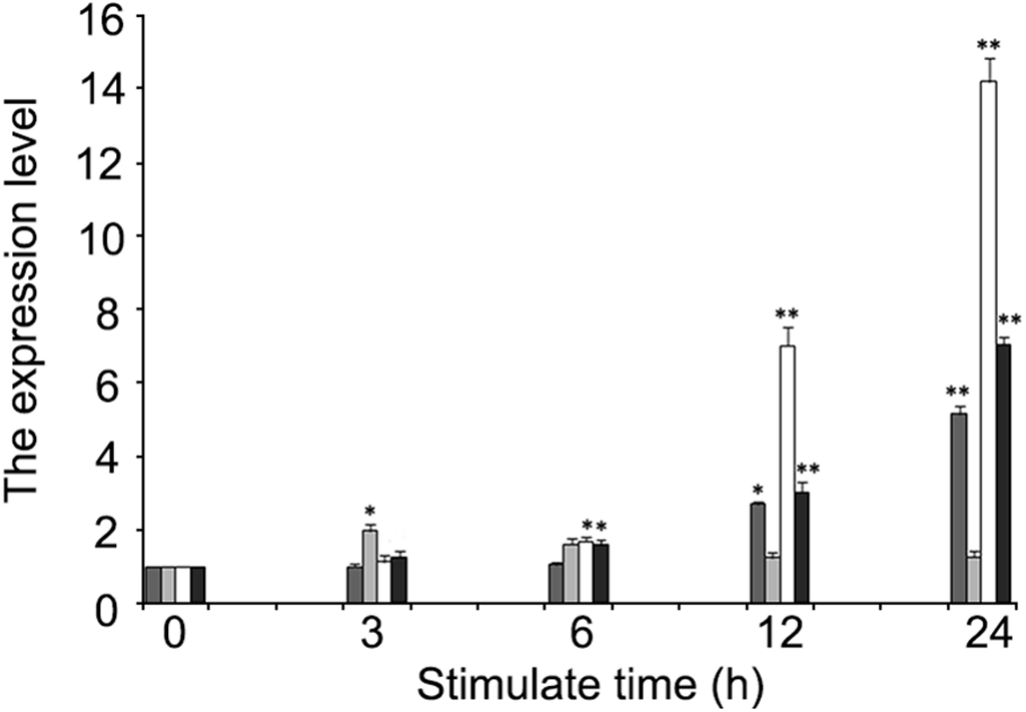
**qRT-PCR of mRNA expression of immune-related genes in EK cells at 3, 6, 12 and 24 h after poly (I: C) exposure, *β*-actin was used as an internal control.** Analysis on the mRNA expression of interferon regulatory factor-7 (black), interferon regulatory factor-3 (white), transforming growth factor-β (light grey) and cytochrome P450 (CYP19A1) (dark grey) in EK cells after immune stimulation are shown; each experiment has been repeated at least three times. The values are displayed as mean±s.e.m. (*P<0.05; **P<0.01; *n*=3).

After poly (I: C) exposure, the expression of TGF-*β* transcripts reached a peak at 3 h with an increased ratio of 1.99-fold (*P*<0.05), and then recovered to the initial level. The expressions of *irf*3 and *irf*7 were significantly upregulated at 6 h post stimulation (*P*<0.05), and showed the maximum increase ratio of 14.18-fold and 7.06-fold at 24 h, respectively (*P*<0.01). The expression of *CYP*450 was increased gradually and presented a significant difference at 12 h, while the peak was observed at 24 h with an increased ratio of 5.15-fold (*P*<0.01) (Fig. 6).

The internal control gene *β*-actin showed no significant change.

## DISCUSSION

EK is the first reported visceral cell line of *A.anguilla* (Fryer and Lannan, 1994; Lakra et al., 2011). As a European catadromous teleost (Schmidt, 1923), the artificial reproduction of *A. anguilla* is still an unresolved question to date, while the wild embryo or neonatal leptocephalus, or even the glass eels, are also extremely difficult to obtain (Huang and Chen, 1998). Establishment of new continuous cell lines should benefit the researches and protection of critically endangered (CR) animal.

Most of the fish cell cultures use mediums developed for mammalian cells, such as DMEM, Ham's F-12, RPMI-1640, L-15, etc. As an amino acid-rich nutrient medium forming a CO_2_-free system, L-15 has been used for successful application on fish cell lines and made CO_2_ incubators unnecessary, which in turn significantly improved the stability and convenience of cell culture (Leibovitz, 1963, 1977). Due to this advantage, more than 80% of the cell lines established after 1994 used Leibovitz's L-15 medium (Lakra et al., 2011). This experiment has proved that L-15 was suitable for European eel tissue and cell culture as well, and this conclusion has been also evidenced by another study on pectoral fin cells of *A. anguilla* (Mao et al., 2012) and the studies on *A. rostrata* cell lines (Bloch et al., 2016; Dewitte-Orr et al., 2006).

Fish cell culture has a convenient temperature range wider than mammalian cell culture, and the EK cells were accustomed to a wider temperature window ranging from 15–37°C. The maximum growth rate was observed at 30°C, but the EK cells under this temperature usually were overgrown in 48 h, and the confluent cell layer would be destructed by contraction and cell detachment in 60 h (Fig. S2). The flow cytometer records proved that suspension cells contributed to the total growth significantly at 30°C or higher temperatures. To meet the needs of regular physiological and pathological experiments, the optimal culture temperature of EK cells was designated 26°C, which was the same as for the *A. japonica* cell line EP-1 (Kou et al., 1995) and the *A. rostrata* cell line eelB (Bloch et al., 2016), and simiar to the *A. rostrata* cell line PBLE (28°C) (Dewitte-Orr et al., 2006). The appropriate temperature range for *A. anguilla* in the culture ponds is 20–26°C (Huang and Chen, 1998).

The karyotype analysis revealed that only 38% of the passage 63 EK cells possessed the modal diploid chromosome number of 2*n*=38 (Yang et al., 1999). The EK strain showed the property to form neoplasms *in vitro*, and its aneuploidy suggested the possibility of immortality (Chen et al., 2005). This may be further tested by telomerase activity analysis (Bryson et al., 2006).

Iridoviruses are common pathogens detected in *A. japonica* (Sorimachi and Egusa, 1987) and other aquaculture species in Fujian province (Yang, 2013). Compared with the former test using the EO cell line (Ge et al., 2012), in which only vesiculation was observed within 1 week, the infection course took much less time on EK cells. The susceptibility of the EK cell line has made it an efficient tool for studying the local viral diseases.

In recent years, cell lines have turned out to be viable tools for function analysis of fish innate immune genes (Poynter and DeWitte-Orr, 2015). Although the rapid responding kinetics of the expression of various immune factors to *in vitro* or *in vivo* inflammatory stimulation has been confirmed repeatedly in the teleosts (Haddad et al., 2008; Huang et al., 2014; Maehr et al., 2013; Sudhakumari et al., 2005), the mechanism of inflammation-induced immune modulation still remained ambiguous. The interferon regulatory factor (IRF) family members played critical roles in cellular differentiation of hematopoietic cells, and the regulation of gene expression in response to pathogen-derived danger signals to regulate cell cycle and apoptosis (Tamura et al., 2008). *Irf*3 was once reported to be upregulated in both peripheral blood leukocytes and *in vivo* after poly (I: C) stimulation in *A. Anguilla* (Huang et al., 2014), and this has been confirmed in this study in association with the changes of *irf*7 levels. The *CYP* gene superfamily consists of a large number of genes encoding *P450* enzymes involved in the detoxification of exogenous chemicals (e.g. drugs, chemical carcinogens, environmental pollutants) and the metabolism of various endogenous substrates (steroids, fatty acids, vitamins, prostanoids, etc.) (Uno et al., 2012). Since the 1980s, multiple forms of *CYP*s have been considered common biomarkers in assessing the contamination of the aquatic environment (Bugiak and Weber, 2009; Nilsen et al., 1998; Winston et al., 1988). In this study, the expression of *CYP*450 showed a significantly long-term upregulation after poly (I: C) induction, indicating the potential of EK cell line for toxicological and pharmacological analysis of aquatic pollutants.

In summary, a continuous cell line from *A. anguilla* kidney has been established and showed its potential impact for studying infectious viral diseases of *Anguillidae* fishes and for immune genetic, toxicological and pathological researches, benefitting the protection of *A. anguilla.*

## MATERIALS AND METHODS

### Primary cell culture and subculture

Healthy *A. anguilla* (elvers) with an average weight of 2 g from an eel farm in Changle, Fujian, China were kept in clean sea water at room temperature (25–28°C) during transportation. Before dissection, the fish were killed by anesthesia and then disinfected by 2% iodine tincture and 75% alcohol three times, respectively. The kidney tissue of the elvers was removed completely, washed five times with 0.01 mol l^−1^ PBS containing 200 IU ml^−1^ of penicillin and 200 μg ml^−1^ of streptomycin (Sangon) at 0°C, and then minced thoroughly into pieces (c. 1 mm^3^). The tissue fragments were rinsed three times with PBS, then attached to the bottom of 25 cm^2^ culture flasks (Corning), and wetted with serum-free Leibovitz’ L-15 (Hyclone) at an interval of 0.5 cm. The full culture medium included L-15 supplemented with 15% FBS (Gibco) and antibiotics as mentioned above. Every single flask was incubated upside-down at 20°C for 6–8 h first. Then 2 ml full culture medium was dripped in before it was overturned to make the tissue explants soaked and 1 ml additional culture medium was added every day till the total volume reached 5 ml. All the flasks were then transferred to 26°C and the culture medium was half-changed every 3 days.

When the radial outgrowths surrounded the tissue explants, the culture was digested with 0.25% trypsin solution (Sigma-Aldrich) at 26°C. After centrifugation at 1000 rpm for 3 min, the cells were collected and suspended in 5 ml full culture medium, then inoculated into a new 12.5 cm^2^ flask (BD falcon) incubated at 26°C until a cell monolayer was formed.

### Cell line development and storage

When a confluent cell monolayer was observed at 26°C, the cells were washed and digested by 0.25% trypsin solution for 1 min, then inoculated to new 25 cm^2^ flasks with a split ratio of 1:2. From the sixth subculture, the antibiotics were no longer additives, and the concentration of FBS was reduced to 10% in the eighth subculture (Lannan, 1994). After 72 h growth *in vitro*, the EK cells were harvested and re-suspended in L-15 containing 20% FBS and 10% dimethyl sulphoxide (DMSO, Sigma-Aldrich) at a density of 10^6^ cells ml^−1^. The cell suspension was dispensed into 1.2 ml cryogenic vials (Corning) and kept initially at 4°C for 30 min, then at −75°C overnight, and finally transferred into liquid nitrogen (−196°C). Thirty days post cryopreservation, the frozen cells were resuscitated in the water bath at 37°C for 2 min, and then suspended and inoculated into 25 cm^2^ cell culture flasks at 26°C, wherein a full medium-change in 12 h was necessary. The cell viability was evaluated by Trypan Blue staining (Ott, 2004).

### Growth studies

Passage 63 EK cells were inoculated to 25 cm^2^ flasks (2×10^5^ cells per flask) and incubated at 15°C, 20°C, 25°C, 30°C and 37°C, respectively. Three flasks of cells from each group were harvested and counted by a flow cytometer every day until day 9. The cell PDT was calculated using the below formula (Davis, 2001):(1)
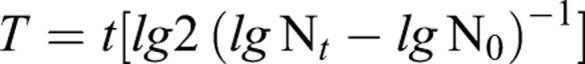


### Chromosome analysis

For chromosome analysis, EK cells at passage 58 were transferred into a 75 cm^2^ culture flask and kept at 26°C for 36 h, and then transferred into L-15 medium containing 2% FBS and 0.5 μg ml^−1^ Colchicine (Sigma-Aldrich). After incubation for 5 h, the cells were collected with centrifugation and treated with 5 ml of 0.3% KCl for 25 min. The cells were prefixed for 5 min by dropping 1 ml of Carnoy's fixative (methanol:acetic acid=3:1, 0°C) into the suspension. After centrifugation at 1500 rpm for 5 min, the cell pellets were fixed with 2 ml Carnoy's fixative for 10 min. The fixed cells were centrifuged and re-suspended in 2 ml Carnoy's fixative, and then incubated at 4°C overnight. The suspension was dropped on cold glass slides, which were tapped to scatter the samples equally. The slides were then air dried and stained with 10% Giemsa (pH 6.8) for 1 h. Under a Nikon Eclipse TE2000-S fluorescence microscope, 100 metaphase cells were photographed and analyzed (Levan et al., 1964).

### Pegfp-N1 transfection

The pEGFP-N1 plasmid (Takara) expressing a green fluorescent protein (GFP) was used for cell transfection. The EK cells at passage 60 were inoculated at a density of 1×10^5^ cells well^−1^ in a 6-well plate. After 48 h, the cells were transfected with 5 μg pEGFP-N1 plasmid in 12 μl lipofectamine™ 2000 reagent (Takara) and incubated at 28°C for 8 h. The cell culture was then transferred to L-15 supplemented with 10% FBS. After 18 h, the fluorescence signals from the cells were observed by a Nikon TE2000S fluorescence microscope, the transfection efficiency was calculated by counting the ratio of GFP-positive cells to all cells in 10 different optical fields.

### *18s* rRNA sequence analysis

For authentication regarding the origin of the EK cell line, its *18s* recombinant (r)RNA gene was sequenced (Englisch, 2001; Frankowski and Bastrop, 2010). The total genomic DNA of passage 45 EK cells was extracted using the Gene JET Genomic DNA Purification Kit (Thermo Fisher Scientific). The fragments of *18s* rRNA gene were amplified using a pair of specific primers (Table 1) designed according to the published total *A. anguilla* *18s* rRNA sequence (GenBank accession no: FM946070.1). The PCR amplification system was composed of a 50 μl reaction mix containing 5.0 μl of 10×buffer, 4.0 μl of deoxynucleotide triphosphate (dNTP) mix (2.5 mM each), 2.0 μl of each primer (10μM each), 0.5 μl of EX-*taq* DNA Polymerase (5 U μl^−1^; Takara) and 2 μl of the extracted genomic DNA. The optimum conditions for PCR include: initial denaturation at 94°C for 5 min, 30 cycles at 94°C for 30 s, 55°C for 1.5 min and 72°C for 1 min, with a final elongation at 72°C for 10 min. The PCR products (5 μl per sample) were collected with High Pure PCR Product Purification Kit (Roche) and analyzed in 1% agarose gels containing 0.5 mg ml^−1^ ethidium bromide, and then photographed using a FR-980A Gel image analysis system (FURI). The final PCR products were sequenced by Shanghai Sangon Biological Engineering Technology & Services Co., Ltd.

**Table 1.**
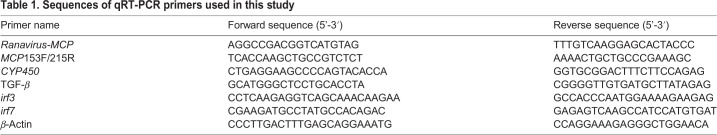
**Sequences of qRT-PCR primers used in this study**

### Quantitative real-time PCR for RGV detection

The primers specific for RGV major capsid protein (MCP) gene amplification and qRT-PCR were synthesized by Shanghai Sangon Biological Engineering Technology & Services Co., Ltd (Table 1). The total DNA of the positive control cells was isolated using Gene JET Genomic DNA Purification Kit (Thermo Fisher Scientific). The MCP gene 695 bp segment was amplified with a 50 μl PCR reaction mix containing 5.0 μl of 10×*Taq* buffer (20 mM Mg^2+^ included), 4.0 μl of deoxynucleotide triphosphate (dNTP) mix (2.5 mM each), 2 μl of each primer, 2 μl of DNA templates, 0.5 μl Ex*Taq* (5 U μl^−1^, Takara) and ddH_2_O.The cycling conditions were as follows: 95°C for 3 min, then 30 cycles at 94°C for 30 s, 55°C for 30 s, 72°C for 35 s. The PCR products were collected and purified using 2% agarose gel and the SanPrep Column DNA Gel Extraction Kit (Sangon). The MCP segment was ligated to the pMD19-T vector with a pMD-19T vector cloning kit (Takara), and expressed in *E. coli* DH5*α* strain. The concentration of the amplified plasmid was measured with a NanoDrop 2000 spectrophotometer (Thermo Fisher Scientific), and converted to the copy number. The plasmid sample was diluted to 10^10^−10^1^ copies μl^−1^ for establishing the standard curve. The qRT-PCR was carried out with an Applied Biosystems®7500 Real-Time PCR System (Thermo Fisher Scientific) using a SYBR® PremixEx Taq*^T M^* II kit (Takara). The standard amplification reaction was performed under the following conditions: 95°C for 30 s, followed by 40 cycles of 95°C for 50 s, 60°C for 34 s; and the dissociation curve determination conditions were as follows: 95°C for 15 s, 60°C for 60 s, 95°C for 15 s.

### Susceptibility test

The susceptibility of EK cells to RGV was investigated. Purified viral samples were prepared according to the previous study (Liu et al., 2012; Ge et al., 2014). The EK cells at passage 45 were inoculated into 25 cm^2^ culture flasks and incubated at 26°C for 48 h, then washed with 0.01 mol l^−1^ PBS. 1 ml of virus suspension (dilution=10^−1^) was added to each flask respectively and removed after 2 h incubation. The infected cells were kept at 26°C in L-15 supplemented with 2% FBS, and observed for a cytopathic effect (CPE) daily under a Nikon ECLIPSE TE2000-S fluorescence microscope. Total DNA was extracted from the cells at 0, 6, 12, 24 and 48 h after infection with Gene JET Genomic DNA Purification Kit, and used as the templates for qRT-PCR. The virus copy number was calculated with the CT standard curve.

### Immune-related gene expression after poly (I: C) exposure

To define the responses of this cell line to immune stimulations, the changes of the expression of interferon regulatory factor-7 (*irf*7, GenBank accession no. KF577784.1), interferon regulatory factor-3 (*irf*3, GenBank accession no. KF577783.1), transforming growth factor-*β* (*TGF*-*β*, GenBank accession no. AJ318934.1) and cytochrome P450 (*CYP*19A1, GenBank accession no. KF990052.1) in the EK cells caused by poly (I: C) induction were detected using qRT-PCR (Wang et al., 2014). Poly (I: C) (final concentration=10 μg ml^−1^; Sigma-Aldrich) was added to the culture medium, and the cells were collected after 3, 6, 12 and 24 h incubation. The total RNA was extracted using a TRIzol®Plus RNA Purification Kit (Invitrogen), and then reverse transcribed into first-strand cDNA as the template for qRT-PCR with SuperScript™ III First-Strand Synthesis Super Mix for qRT-PCR (Invitrogen). With the primers designed by Primer Premier 6.0 and Beacon designer 7.8 (Table 1), qRT-PCR was performed in the CFX384 Touch™ Real-Time PCR Detection System (Biorad) using the Power SYBR® Green PCR Master Mix reagent (Applied Biosystems). The conditions of reaction system were as follows: a 20 μl reaction mix containing 8.0 μl of ddH_2_O, 10.0 μl of Power SYBR^®^ Green PCR Master Mix, 0.5 μl of each primer and 1.0 μl of first-strand cDNA, while the cycling conditions were as follows: initial temperature at 95°C for 1 min, then 40 cycles at 95°C for 15 s, 63°C for 25 s. The 2^−ΔΔct^ method was used to analyze the relative expression level.

### Statistical analysis

Each experiment was repeated at least three times. The data are shown as mean_±_s.e.m._,_ and the statistical significance was determined using one-way analysis of variance (Dunnett's T3 test) (Davis, 2001). Statistical analysis was done using SPSS (www.ibm.com/analytics/).

## Supplementary Material

Supplementary information
