# Supplementary material for: Development and characterization of a new cell line derived from European eel Anguilla anguilla kidney
Source: Biol Open. 2018 Nov 14;8(1):bio037507. doi: 10.1242/bio.037507 (PMC6361207; doi:10.1242/bio.037507)
Supplement: Supplementary information [file biolopen-8-037507-s1.pdf]

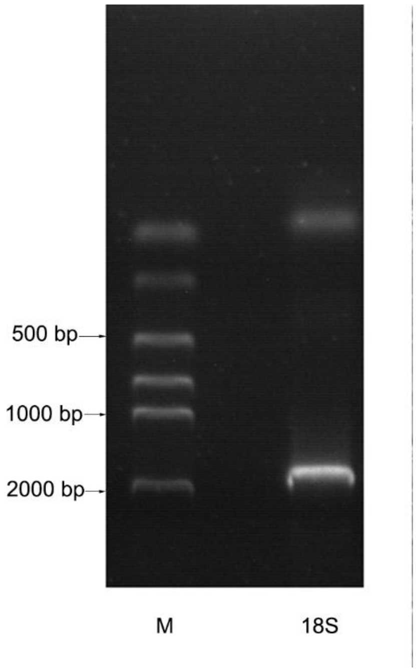

Fig. S1. Species authentication. Agarose gel electrophoretic pattern of *18s* rRNA of the EK cells.

Biology Open • Supplementary information

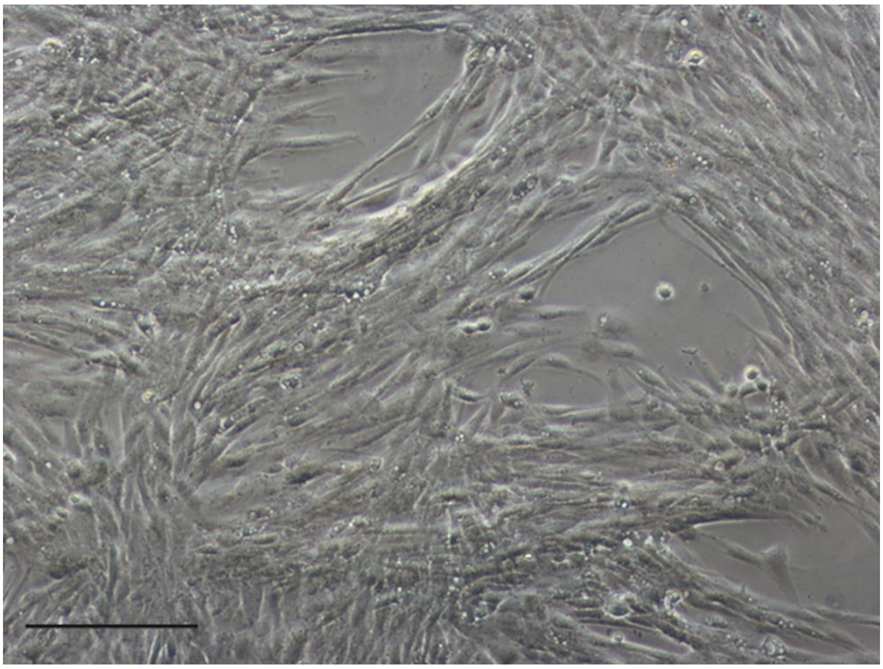

Fig. S2. Overgrowth and detachment of EK cells at 30°C. passage 63 EK cells, 60 h after subculture. Scale bars = 50µm.
